# In vivo quantification of blood mixing in single ventricle patients with Fontan circulation using 4D flow MRI

**DOI:** 10.1186/1532-429X-15-S1-E88

**Published:** 2013-01-30

**Authors:** Kelly B Jarvis, Susanne Schnell, Maya Gabbour, Alex J Barker, Ramona Lorenz, James Carr, Joshua D Robinson, Andrada R Popescu, Roger A de Freitas, Cynthia Rigsby, Michael Markl

**Affiliations:** 1 Radiology, Northwestern University Feinburg School of Medicine, Chicago, IL, USA; 2 Medical Imaging, Ann & Robert H Lurie Children's Hospital of Chicago, Chicago, IL, USA; 3 Pediatrics, Northwestern University Feinberg School of Medicine, Chicago, IL, USA; 4 Division of Cardiology, Ann & Robert H Lurie Children's Hospital of Chicago, Chicago, IL, USA; 5 Radiology, University Medical Center Freiburg, Freiburg, Germany

## Background

Single ventricle physiology (SVP) is one of the most severe forms of complex congenital heart disease (CHD). Patients undergo multiple surgical interventions including the Fontan procedure (caval venous return is routed directly to the pulmonary arteries). Despite the apparent success of the procedure, it is unclear why some patients develop ‘failing Fontan physiology' while others remain asymptomatic. However, there is growing evidence that underlying hemodynamics in the Fontan circulation may play an important role [1]. Uneven distribution of blood from the caval to the pulmonary system has been suspected to influence patient outcome through the delivery of protein-rich venous return [2]. Therefore, the aim of this study was to employ whole heart 4D flow MRI to visualize and quantify ‘blood mixing at the Fontan connection' (upper and lower venous blood distribution to the LPA and RPA).

## Methods

4D flow MRI (spatial resolution = 2.5-3.8 x 2.5-3.3 x 2.5-3.3 mm^3^, temporal resolution = 37.6-40.8 ms) with whole heart coverage was performed at 1.5T and 3T systems (Trio, Avanto, Siemens, Germany) in 8 patients (3 females 5 males, age 17 +/- 6, range 5-26) with Fontan circulation. Time-averaged 3D phase contrast angiograms (PC-MRAs) were calculated using 4D flow MRI data to depict the Fontan vascular geometry. Time-resolved particle pathlines were generated from analysis planes in the caval veins to illustrate the spatial distribution and dynamics of blood flow to the left and right lungs (EnSight, CEI, USA). Blood mixing was quantified by counting the number of pathlines reaching analysis planes in the RPA and LPA (Matlab, The MathWorks, USA). In addition, SVC-IVC offsets were estimated as a measure of Fontan geometry.

## Results

**Figure 1:**
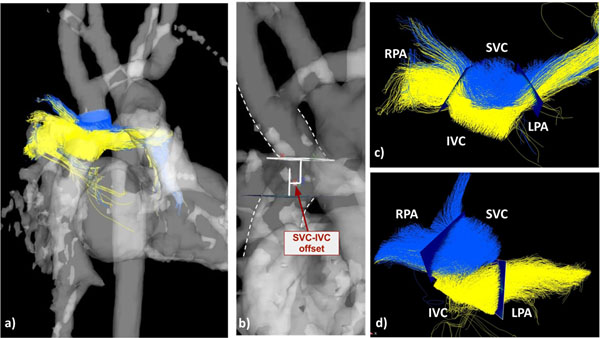
The 3D PC-MRA was used to manually position four analysis planes in the Fontan pathway: superior vena cava (SVC), inferior vena cava (IVC), left pulmonary artery (LPA) and right pulmanary artery (RPA). Pathlines were generated from the IVC and SVC and color coded by vessel of origin. For an example patient, the a) PC-MRA and pathlines, b) SVC-IVC offset estimation and c) plane placement for mixing analysis are shown. The d) blood flow pathlines and plane placement for another patient with asymmetric flow distribution are shown for comparison.

3D visualization and quantification results for blood mixing at the Fontan connection varied substantially between patients (Figure 1, Table 1). Linear regression analysis revealed a strong correlation between the asymmetry of flow distributions to the RPA and LPA (% difference in pathline distributions to RPA and LPA) and SVC-IVC offsets for both the SVC (r=0.72, p=0.04) and IVC (r=0.79, p=0.02).

**Table 1:** For each patient with Fontan circulation, the number of IVC and SVC pathlines reaching analysis planes in the LPA and RPA were normalized over the total number of pathlines that reached either PA. The percent pathline distribution from the IVC and SVC to the LPA and RPA are shown as well as the SVC-IVC offset estimation. In 5 patients, SVC pathlines indicated flow predominantly to the RPA. In all but one patient, IVC pathlines represented flow predominantly directed toward the RPA.

Patient	Blood mixing						Fontan geometry
	Flow originating in IVC			Flow originating in SVC			

Number	to LPA [%]	to RPA [%]	Diff [%]	to LPA [%]	to RPA [%]	Diff [%]	SVC-IVC offset [mm]

1	12.3	87.7	75.3	60.0	40.0	20.0	5.5
2	21.5	78.5	56.9	45.8	54.2	8.4	5.4
3	40.5	59.5	19.0	40.4	59.6	19.1	1.8
4	25.7	74.3	48.6	30.6	69.4	38.8	2.5
5	28.7	71.3	42.6	71.8	28.2	43.6	3.4
6	14.2	85.8	71.6	93.0	7.0	85.9	10.2
7	85.8	14.2	71.7	0.6	99.4	98.8	8.3
8	1.0	99.0	98.0	21.2	78.8	57.6	7.9

## Conclusions

Using 4D flow MRI, blood flow distribution was shown to vary between patients with Fontan circulation, indicating non-uniformity in the distribution of protein-rich hepatic blood carried in the IVC to the lungs and demonstrating the value of 4D flow MRI for the individual assessment of complex Fontan hemodynamics. These findings also indicate a relationship between Fontan geometry (SVC-IVC offset) and blood distribution to the left and right lungs. This study was limited by the number of subjects and the spatial and temporal resolution. In addition, SVC-IVC offsets are estimates and represent a simplified depiction of the Fontan geometry.

## Funding

Grant support by NIH R01HL115828, NUCATS Dixon Award.
